# Alginate-based hydrogels loaded with human *β*-defensin-2 promote healing of MRSA-infected wounds in a diabetic model: a preclinical proof-of-concept study

**DOI:** 10.1007/s10238-025-01798-6

**Published:** 2025-07-15

**Authors:** Jessica Da Silva, Daniela Calheiros, Teresa Gonçalves, Eduardo A. Silva, Eugénia Carvalho, Ermelindo C. Leal

**Affiliations:** 1 Institute for Interdisciplinary Research, Doctoral Program in Experimental Biology and Biomedicine (PDBEB), University of Coimbra, Coimbra, Portugal; 2 CNC-UC—Center for Neuroscience and Cell Biology, University of Coimbra, Coimbra, Portugal; 3 CIBB—Centre for Innovative Biomedicine and Biotechnology, University of Coimbra, Coimbra, Portugal; 4 Department of Biomedical Engineering, Genome and Biomedical Sciences Facility, UC Davis, 451 Health Sciences Dr., Davis, CA 95616 USA; 5 Faculty of Medicine, Doctoral Program in Health Sciences (PHDHS), University of Coimbra, Coimbra, Portugal; 6 Faculty of Medicine, University of Coimbra, Coimbra, Portugal; 7 Department of Chemistry, Bioscience, and Environmental Engineering, University of Stavanger, Kristine Bonnevies Vei 22, 4021 Stavanger, Norway; 8 Institute for Interdisciplinary Research, University of Coimbra, Coimbra, Portugal

**Keywords:** Antimicrobial peptides (AMPs), Chronic wounds, Diabetic foot infections (DFIs), Multidrug-resistant microorganisms, Sustained release, Wound dressing

## Abstract

Diabetic foot infections (DFIs), resulting from microbial colonization and proliferation in non-healing diabetic wounds, are among the most serious and common complications in patients with diabetes. As antimicrobial resistance continues to rise, the clinical management of DFI persists as a major challenge, emphasizing the need for novel therapeutic approaches. In this study, we aimed to combine the dual antimicrobial and pro-healing properties of antimicrobial peptides (AMPs) with the intrinsic characteristics of the alginate polymer as an encouraging strategy to address the multifactorial etiology of chronic wounds. Using ionic cross-linking with calcium sulfate, we developed alginate-based hydrogels with a nanometric porous structure for the sustained delivery of the AMP human β-defensin-2 (hBD-2) to promote wound healing in conditions of diabetes. The effects of the produced hBD-2 hydrogels were assessed in a streptozotocin-induced diabetic mouse model with wounds infected by methicillin-resistant *Staphylococcus aureus* (MRSA). Overall, hBD-2 hydrogels improved wound closure, by promoting re-epithelialization and tissue remodeling, ultimately restoring normal epidermal thickness. Moreover, hBD-2 hydrogels attenuated the wound MRSA load, while decreasing the inflammatory state. Lastly, hBD-2 hydrogels increased the number of Ki67^+^ cells and CD31^+^ cells, indicating improved cellular proliferation and angiogenesis, ultimately supporting the evidence of an early progression toward the final phases of wound healing. Despite the difficult MRSA-infected wound conditions, the findings underline the potential of hBD-2 hydrogels as a promising treatment for chronic wounds such as DFUs, owing to antimicrobial, anti-inflammatory, and tissue-regenerative properties.

## Introduction

Wound infection is an aggravating issue for diabetic foot ulcers (DFUs), that develops in 50 to 60% of cases [1, 2]. These diabetic foot infections (DFIs) are characterized by a multi-kingdom microbiota, predominantly associated with Gram-positive bacterial colonies, such as *Staphylococcus aureus* (MSSA—methicillin-susceptible *S. aureus* and MRSA—methicillin-resistant *S. aureus*), *Streptococcus β-hemolytic*, and *Corynebacterium striatum*, but also with Gram-negative bacterial colonies, such as *Pseudomonas aeruginosa* and *Escherichia coli*, as well as anaerobes like *Bacteroides* spp. and *Prevotella* spp. frequently present in deeper wound layers [1–3]. Fungal pathogens, such as *Candida* spp. and *Trichophyton* spp., are also often present and have been linked to wound necrosis and poor clinical outcomes, while anaerobic bacteria are associated with non-healing DFUs [3, 4]. These microorganisms can live independently or organize into functionally equivalent pathogroups, where commensal and pathogenic microorganisms co-aggregate symbiotically in a pathogenic biofilm for more efficient nutrient cycling and enhanced protection from external threats, further promoting chronic infection [3]. The development of DFIs is influenced by the overall microbial load, species diversity, presence of pathogenic microorganisms, and synergistic association among microbial species [5]. Indeed, the wound microbiota and the presence of microbial biofilms are key players of impaired healing in chronic wounds. Therefore, the control of pathogen infection is critical to ameliorate the complex microenvironment of DFUs and subsequently promote a continuous healing over time [6].

Current management of DFUs relies on a multifaceted approach, including metabolic control, foot assessment, surgical debridement, control of infection with antibiotics, dressing application, offloading pressure, referral to multidisciplinary teams, and patient education on DFU health literacy [7]. Nonetheless, the increasing prevalence of multidrug-resistant (MDR) microorganisms has limited the treatment efficacy of DFIs and increased the number of prolonged hospitalizations and subsequent amputations. For instance, a small prospective study involving 41 patients with DFU revealed that 73% had infected ulcers, 70% of which were polymicrobial and displayed a high prevalence of MDR pathogens, particularly of MRSA, underlining the occurrence of multiple hospitalizations [8]. Another meta-analysis study further established MDR bacterial infection as an important risk factor for DFU recurrence [9].

In this context, antimicrobial peptides (AMPs) appear as promising alternatives to conventional antibiotics. AMPs exert broad-spectrum antimicrobial activity via membrane dysruption on pathogens, in contrast to most antibiotics that target specific proteins, thus reducing the risk of microbial resistance development [10]. However, despite their encouraging dual broad-spectrum antimicrobial and multi-targeted wound healing activities, AMPs may present some limitations, including low stability and potential toxicity, on top of impaired expression level and functionality in diabetic conditions [6]. To overcome these challenges, biomaterials as biocompatible delivery systems for exogenous AMPs have emerged as a promising approach [11].

In this work, we focused on the alginate polymer, which offers several benefits as a wound dressing for the delivery of AMPs. In detail, the anionic nature of alginate is associated with excellent biocompatibility, hydrophilicity, and great absorbing capacity, all representing appropriate features for wound healing applications [12, 13]. Moreover, alginate-based dressings form a protective barrier that maintains suitable moist content and temperature for wound healing, while also enabling ion exchange with wound exudate and blood [14, 15]. Finally, when used as a delivery platform, alginate can also protect AMPs from proteolytic degradation and enable their sustained release over time, enhancing their bioactivity and therapeutic potential [16].

Herein, we settled an alginate-based hydrogel with a nanoporous structure for the sustained release of the AMP human β-defensin-2 (hBD-2), aimed at promoting healing for MRSA-infected wounds in conditions of diabetes, likely ameliorating the quality of life of patients with DFIs and minimizing the associated socioeconomic burden. This novel therapeutic approach has already been validated in preclinical in vitro and in vivo models from our group [17] and is now being evaluated in an in vivo diabetic mouse model of MRSA-infected wound healing as described below. In this study, we established a diabetic mouse model with long-term hyperglycemia and wound infection using a clinical MRSA strain to better mimic the chronicity and complexity of human DFIs. Few studies to date have considered diabetes and infection in their in vivo models of wound healing [18–21], and even fewer have been performed regarding the chronicity of diabetes and the complexity of wound infection, hence not adequately resembling the human condition of DFI [22]. Our model allows for a more accurate assessment of novel biomaterial-based therapeutic approaches and holds potential for improved translational relevance into DFI clinical practice.

## Materials and methods

### Materials

Alginate polymers were purchased from NovaMatrix, Norway. Furthermore, the AMP hBD-2 was purchased from ProSpec-Tany TechnoGene Ltd, Israel.

The primary antibodies anti-rabbit interleukin-6 (IL-6) and anti-rat Kiel 67 (Ki67) were purchased from Thermo Fisher Scientific, MA, USA, whereas the primary antibodies anti-rat CD31 and anti-rabbit matrix metalloproteinase-9 (MMP-9) were obtained from Millipore, MA, USA. Moreover, the primary antibodies anti-rat CD206 and anti-goat collagen type 1 alpha 1 (COL1A1) were obtained from Santa Cruz, CA, USA. Furthermore, the primary antibody anti-rat tumor necrosis factor alpha (TNF-α) was purchased from Bio-Rad AbD Serotec Ltd, CA, USA, whereas the primary antibodies anti-rabbit CD3 and CD68 were purchased from Abcam Plc, Cambridge, UK. In turn, the primary antibody anti-rabbit myeloperoxidase (MPO) was acquired from Upstate Biotechnology Inc., NY, USA. Additionally, the normal goat serum was obtained from Life Technologies, CA, USA. The secondary antibodies goat anti-rat Alexa Fluor 594, anti-rabbit Alexa Fluor 488, and anti-rabbit Alexa Fluor 594, as well as the secondary antibody rabbit anti-goat Alexa Fluor 488, were obtained from Abcam Plc, Cambridge, UK. The DAPI (4′,6-diamidino-2-phenylindole) and dihydroethidium (DHE) probes were acquired from Sigma-Aldrich, MO, USA.

All the remaining reagents were purchased from Sigma-Aldrich, MO, USA or VWR, Portugal.

### Hydrogel formulation and loading

Hydrogels were prepared using MVG alginate containing a higher G-block content (> 60% as specified by the manufacturer), including a high molecular weight (HMW) LF20/40 and a low molecular weight (LMW) LF10/60 polymer. The molecular weights of the HMW and the LMW polymers are of ~ 250 kDa and ~ 120–150 kDa, respectively. Alginate polymers used for in vitro and in vivo assays were prepared under sterile and aseptic conditions.

For the formulation of the hydrogels, LMW and HMW alginates were reconstituted in ultrapure water to a final concentration of 3% (w/v) polymer alginate solution. The LMW and HMW alginate solutions were then mixed within interconnected syringes in a proportion of 75/25 (LMW/HMW), followed by a mixture with a gelling agent, a calcium sulfate slurry at a ratio of 4:5, to ionically cross-link the polymer chains. The mixture was rapidly dispensed onto a glass plate set with 1-mm spacers, sandwiched with another glass plate, and incubated for at least 25 min at room temperature. Afterward, disks were punched out using a 6-mm-diameter biopsy punch (Utilmédica—Produtos Médicos Hospitalares, Portugal), and incubated in phosphate buffered saline (PBS) containing calcium and magnesium ions for at least 3 h at 37 °C, 5% CO_2_ to allow swelling and stabilization. For the loading of AMPs onto the 6-mm-diameter hydrogels, the AMP hBD-2 was previously added to the syringe with the LMW alginate solution to a final concentration of 1 µg/mL. A new set of hydrogels was prepared for each experiment.

### In vivo diabetic mouse model of infected wound healing

Twenty-four eight-week-old male C57BL/6 J mice (20–30 g, Charles River Laboratories, Saint Germain Nuelles, France) were acclimated for two weeks under standard housing conditions (12 h light/dark cycle, water and pellet food ad libitum). Prior to wounding and inoculation procedures, animals were transferred to an Animal Biosafety Level 2 area and individualized in separate cages. All the experimental protocols involving animals were approved by the animal research ethics committee of the Center for Neuroscience and Cell Biology (ORBEA_213_2019/28082019) and the Faculty of Medicine of the University of Coimbra and conducted in accordance with the European Directive 2010/63/EU and the Portuguese Decree-Law (113/2013) for the use of animals in scientific research.

Type 1 diabetes mellitus was induced by low-dose intraperitoneal injection of streptozotocin (STZ, 50 mg/kg) in a saline solution, for five consecutive days, as previously described [23]. Mice were considered diabetic when blood glucose levels, measured by the Accu-Check Aviva glucometer one week after the last STZ injection, were higher than 250 mg/dL. The animals were maintained diabetic for a period of 6 to 8 weeks prior to the wound induction procedure to mimic chronicity of the condition. During this period, blood glucose levels were regularly checked, and insulin (1–2 IU/mL) was administered via intraperitoneal injection if needed to avoid weight loss.

**Fig. 1:**
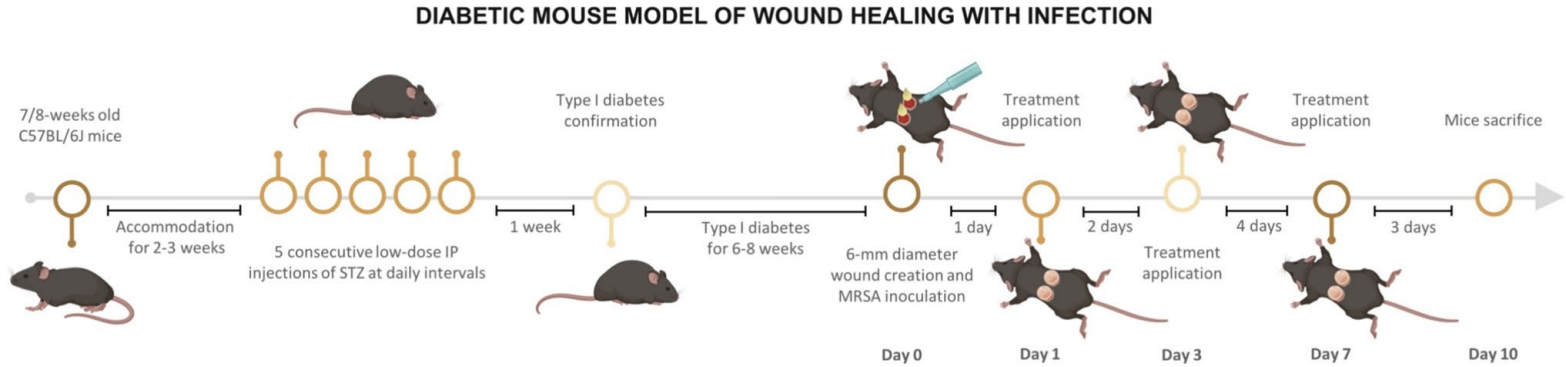
Protocol and respective timeline for establishment of the chronic diabetic mouse model of wound healing with infection

Mice received a subcutaneous (SC) injection of buprenorphine (0.05 mg/kg) 30 min before surgery for mitigation of pain due to posterior surgical procedures. After this time, animals were anesthetized with isoflurane, and the dorsal hair of the mice was shaved and then removed with a depilatory cream. This region was then carefully cleaned with water and 70% ethanol, and a povidone-iodine antiseptic solution was locally applied before the surgical procedure. Afterward, two 6-mm-diameter full-thickness wounds were created with a biopsy punch in each animal and inoculated with 10^8^ colony-forming units (CFUs)/wound of *S. aureus* (DSM113531—clinical MRSA strain isolated from a deep wound in lower leg). Wounds were then covered with Tegaderm (3 M Health Care). Immediately after the surgical procedure, mice received an additional SC injection of buprenorphine (0.05 mg/kg), followed by 0.1 mg/kg every 6–8 h for 48 h, as need for postoperative pain relief. A schematic protocol overview is shown in Fig. 1.

After 24 h of wound inoculation with MRSA, the twenty-four animals were randomly assigned to four experimental groups according to the different wound treatments (n = 6 per group): control—gauze moistened with 28 µL of a saline 0.9% NaCl solution; hBD-2–28 µL of saline 0.9% NaCl solution containing 1 µg/mL of the AMP hBD-2 covered with a gauze; blank hydrogels—6-mm-diameter hydrogel disks with no loaded cargo; and hBD-2 hydrogels—6-mm-diameter hydrogel disks loaded with 1 µg/mL of the AMP hBD-2. Treatments were covered with Tegaderm and reapplied on days 3 and 7. Animals were sacrificed at day 10, then the wounded skins were harvested and included in optimal cutting temperature (OCT) gel and frozen in dry ice or incubated in buffered 4% paraformaldehyde. The number of mice per experimental group was determined based on the following power analysis: equal sample size and variability (13% relative to the mean) among the groups, at *p* < 0.05, with 80% power and 25% expected difference in wound size between groups, being found to be n = 6 per experimental group.

### Wound closure

The progress of wound healing was evaluated by acetate tracing directly on top of each wound over Tegaderm (with duplicates for each wound) and corroborated by photograph acquisition at days 0, 3, 7, and 10. Automated wound size quantification was then performed from the acetate measurements using the image processing Fiji software (Fiji is just ImageJ2 version 2.9.0/1.53t).

### Microbial load

Sterile swabs moistened with a saline solution were applied to each wound for 30 s on days 1, 3, 7, and 10, to collect the associated microbiota. The swabs were transferred to 2-mL eppendorf tubes with 500 µL of saline solution, well squeezed to release the content, and 10 µL of the obtained suspensions was plated in petri dishes containing tryptic soy broth solid medium to allow microbial growth, in an incubator at 37 °C, for 7 days. On day 7, the number of colonies was counted, and the microbial load was expressed as the number of CFUs/mL of suspension relative to the number of CFUs/mL of suspension on day 1 (after 24 h of infection development).

### Histopathological analysis

Paraffin-embedded tissue Sects. (5 µm thickness) were stained using hematoxylin and eosin (Thermo Fisher Scientific, MA, USA) or Masson–Goldner’s trichrome staining kits (Carl Roth, Germany), according to manufacturers’ protocols. Images were acquired using a Carl Zeiss Axio Imager Z2 upright widefield microscope (10 × objective) with the Zen Blue software (version 2012) and analyzed using the image processing Fiji software (Fiji is just ImageJ2 version 2.9.0/1.53t). Epidermal thickness index (ETI) was calculated as the average thickness of epidermis in wounded skin divided by the average thickness of epidermis in uninjured skin and multiplied by 100, while the epidermal thickness was measured in three different regions of each wound bed and is presented as the average thickness (in µm). Collagen content was expressed as a percentage relative to control wounds. Lastly, wound healing was scored (0–12) using the histology scoring system previously established and described by Vyver et al. [24]. In brief, this scoring system for murine cutaneous wounds evaluates key parameters in each phase of healing to establish an overall histology score, ranging from 0 (open/unhealed wound) to 12 (completely healed wound with no scarring). Those parameters include re-epithelization, epithelial thickness index, keratinization, granulation tissue thickness, remodeling, and scar elevation index.

### Immunohistochemistry

Immunohistochemistry was used to analyze the pattern of several inflammatory cytokines (IL-6) and cells (neutrophils, M1 and M2-like macrophages and T lymphocytes), collagen type I (COL1A1) and other important factors in proliferation (Ki67^+^ cells), tissue remodeling (MMP-9), and neovascularization (CD31^+^ cells). Cryosections (10 µm thickness) were fixed in ice-cold acetone and stained for detection of the above described parameters. Samples were examined using a Carl Zeiss LSM 710 confocal microscope (40 × objective). The image acquisition process was conducted using the Zen Black software (version 2012), while the image analysis process was performed using the image processing Fiji software (Fiji is just ImageJ2 version 2.9.0/1.53t).

### Dihydroethidium assay

Skin samples included in the OCT gel were cut into cryosections with 10 µm thickness using a cryostat. Cryosections (10 µm thickness) were stained with 10 µM DHE in PBS to assess reactive oxygen species (ROS) levels. Samples were observed in the Carl Zeiss LSM 710 confocal microscope (40 × objective). The image acquisition process was conducted using the Zen Black software (version 2012), while the image analysis process was performed using the image processing Fiji software (Fiji is just ImageJ2 version 2.9.0/1.53t). ROS levels were expressed as a percentage of DHE signal relative to the control group.

### Statistical analysis

Normal data distribution was assessed with the Shapiro–Wilk test. To assess effects of time and hBD-2 hydrogels on wound closure and microbial load, groups were compared using a two-way ANOVA followed by Dunnett’s multiple comparisons test. Comparisons between groups to assess the effects of hBD-2 hydrogels on different parameters of wound healing were made using a one-way ANOVA followed by Holm–Sidak’s multiple comparisons test or Kruskal–Wallis test followed by Dunn’s multiple comparisons test, depending on sample distribution. Data are presented as mean ± SD or mean ± SEM for parametric data and median (interquartile range, Q1–Q3) for non-parametric data. All analyses were performed using IBM SPSS version 28 (SPSS Inc., Chicago, IL, USA). GraphPad Prism version 8 (GraphPad Inc., La Jolla, CA, USA) was used for graphical representation. A *p* value of ≤ 0.05 was considered statistically significant.

## Results

### hBD-2 hydrogels improve wound closure in an STZ-induced diabetic mouse model of MRSA-infected wounds

**Fig. 2:**
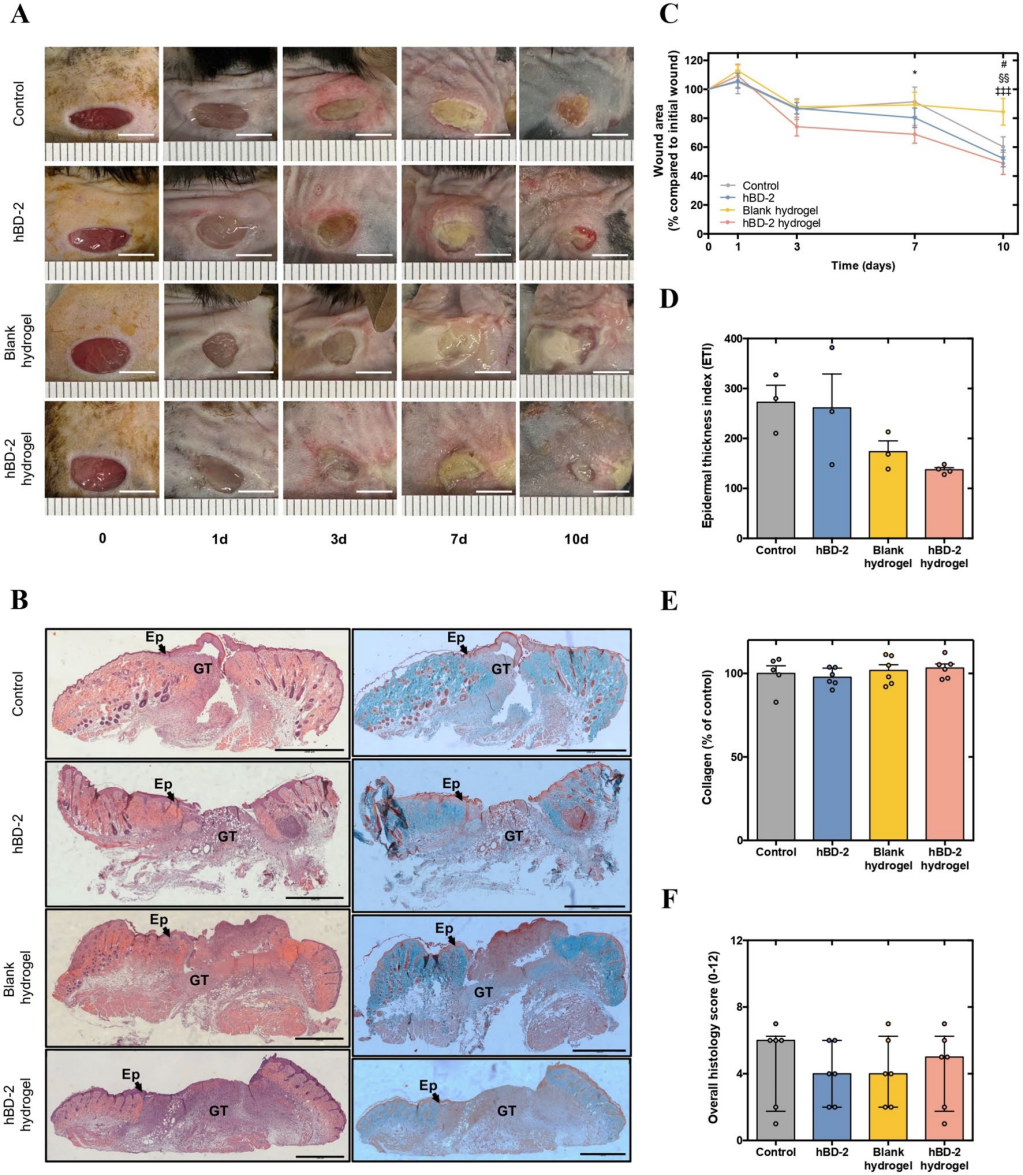
In vivo efficacy of hBD-2 hydrogels in promoting wound healing. **A** Representative images of wound healing progression over time for each group (days 0, 1, 3, 7, and 10). Scale bars—5 mm. **B** Representative histological images of hematoxylin and eosin (left), and Masson’s trichrome (right) for each group. Scale bars—1000 µm. Ep: epidermis; GT: granulation tissue. **C** Wound closure (% of initial wound area) obtained through acetate measurements. **D** Epidermal thickness index calculated as the average thickness of epidermis in wounded skin divided by the average thickness of epidermis in uninjured skin and multiplied by 100. **E** Collagen fiber quantification (% of control) in wounded area. **F** Quantification of the overall histology score. Parametric data (C) are presented as mean ± standard error of the mean (SEM), while histological data (D) and (E) are presented as bars representing mean with individual measurements denoted by scatter points and the error bars indicate standard deviation (SD). Non-parametric data (F) are presented as bars representing median with individual measurements denoted by scatter points and the error bars indicate interquartile range. Statistical analysis was conducted using the two-way ANOVA followed by Dunnett’s multiple comparisons test for wound closure data and the one-way ANOVA followed by Holm–Sidak’s multiple comparisons test or the Kruskal–Wallis test followed by Dunn’s multiple comparisons test, depending on the data distribution, for histological data. Asterisk(s), and hash, section and double dagger sign(s) specify statistically significant differences between the following conditions: * control *versus* hBD-2 hydrogels (*p* < 0.05), # blank hydrogels *versus* control (*p* < 0.05), §§ blank hydrogels *versus* hBD-2 (*p* < 0.01), and ‡‡‡ blank *versus* hBD-2 hydrogels (*p* < 0.001)

After adequate physicochemical characterization and validation using preclinical in vitro and in vivo models of wound healing [17], an STZ-induced diabetic mouse model with MRSA-infected wounds was established to assess the effects of the produced alginate-based hydrogels (blank or loaded with hBD-2) on wound closure and infection control compared to a gauze control and the free hBD-2. The hBD-2 hydrogels significantly accelerated wound closure by day 7 (*p* < 0.05 vs. control) and day 10 (*p* < 0.001 vs. blank hydrogels) (Fig. 2A and C), with improved reepithelialization and early tissue remodeling evident in histological images (Fig. 2B). Besides, hBD-2 hydrogels also induced an overall faster wound closure by more than 1.16-fold from day 3 to 7 compared to the other three experimental groups (Fig. 2A and C). Moreover, both blank and hBD-2 hydrogels decreased the ETI by more than 1.50-fold compared to control and free hBD-2(Fig. 2D). Lastly, no significant changes were detected on collagen fiber quantification nor on histology scores among the experimental groups (Fig. 2E and F).

### hBD-2 hydrogels attenuate the microbial load in skin MRSA-infected wounds of diabetic mice

**Fig. 3:**
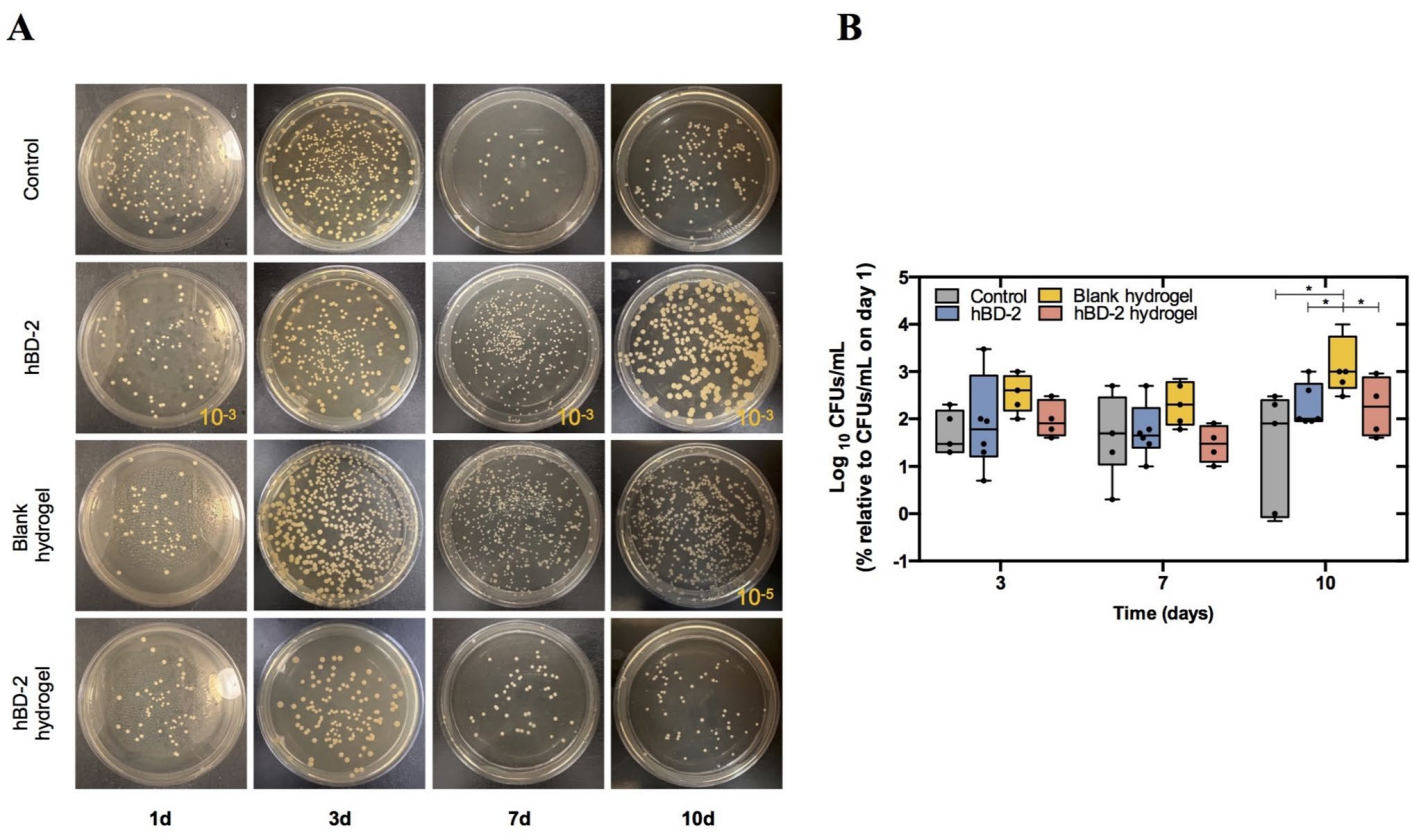
Effects of hBD-2 hydrogels on microbial load in infected wounds. **A** Representative images of wound microbial load over time for each group (days 1, 3, 7, and 10) at dilution = 10.^−4^. **B** Quantification of wound microbial load obtained from topical swab collection relative to wound microbial load on day 1 (after 24 h of infection development). Data are presented as median with interquartile range, individual measurements being denoted by scatter points. Statistical analysis was conducted using the two-way ANOVA followed by Dunnett’s multiple comparisons test. Asterisk(s) specify statistically significant differences between conditions. *p < 0.05

The effects on wound microbiota were assessed after 24 h of MRSA colonization, by topical swab collection on days 3, 7, and 10. Overall, hBD-2 hydrogels led to a decrease on the MRSA load by 3.68-fold and 8.27-fold on days 3 and 7, respectively, compared to blank hydrogels (Fig. 3A and B). Similarly, free hBD-2 led to a decrease on the MRSA load by 2.70-fold on day 7 compared to blank hydrogels (Fig. 3A and B). Besides, both free hBD-2 and hBD-2 hydrogels, as well as the control group, further showed a decrease on the MRSA load on day 10 compared to blank hydrogels (*p* < 0.05) (Fig. 3A and B). However, no significant changes were observed compared to the control group (Fig. 3A and B).

### hBD-2 hydrogels reveal anti-inflammatory properties in skin MRSA-infected wounds of diabetic mice

**Fig. 4:**
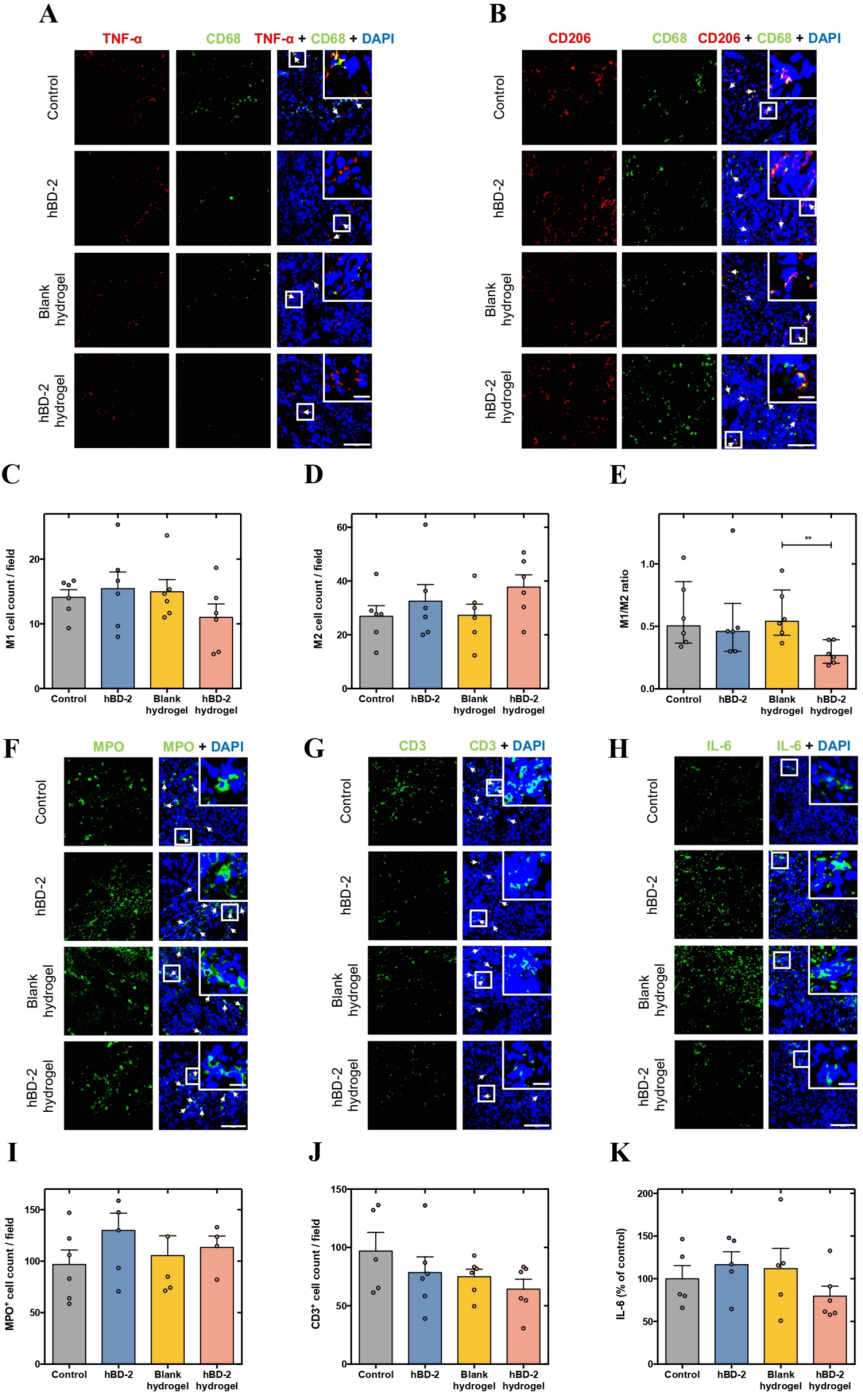
Anti-inflammatory effects of hBD-2 hydrogels at day 10 post-injury/infection. Representative images of **A** M1-like macrophages and **B** M2-like macrophages, both indicated by white arrows. Scale bars—20 µm (picture inlets) and 100 µm. Quantification of **C** M1-like macrophages, **D** M2-like macrophages, and **E** M1/M2 ratio. Representative images of **F** myeloperoxidase (MPO)^+^ cells (indicated by white arrows), **G** CD3^+^ cells (indicated by white arrows), and **H** interleukin-6 (IL-6). Scale bars—20 µm (picture inlets) and 100 µm. **I** Quantification of MPO^+^ cells per field at the wound site. **J** Quantification of CD3.^+^ cells per field at the wound site. **K** Percentage of IL-6 expression levels relative to the control group. Bars represent mean ± standard error of the mean (SEM) for parametric data and median with interquartile range for non-parametric data, both with individual measurements denoted by scatter points. Statistical analysis was conducted using the one-way ANOVA followed by Holm–Sidak’s multiple comparisons test or the Kruskal–Wallis test followed by Dunn’s multiple comparisons test, depending on the data distribution. Asterisk(s) specify statistically significant differences between conditions. **p < 0.01

Several wound inflammation-related parameters were investigated at day 10 post-injury/infection through immunohistochemical analysis of wound tissue to assess the anti-inflammatory potential of hBD-2 hydrogels. In particular, M1 and M2-like macrophages were quantified using CD68 and TNF-α or CD68 and CD206 markers, respectively. A decrease by more than 1.25-fold of M1-like macrophages was observed in the hBD-2 hydrogel group compared to the other experimental groups (Fig. 4A and C). In turn, an increase by more than 1.35-fold of M2-like macrophages was observed in the hBD-2 hydrogel group compared to the control and the blank hydrogel groups (Fig. 4B and D). The free hBD-2 group also increased, yet with a slighter extent (1.19-fold), the number of M2-like macrophages (Fig. 4B and D). Correspondingly, the M1/M2 macrophage ratio was significantly lower in the hBD-2 hydrogel group than in the blank hydrogel group (*p* < 0.01), suggesting a shift toward a pro-regenerative immune profile (Fig. 4E). In addition, neutrophil infiltration (MPO-positive cells) and T lymphocyte presence (CD3-positive cells) showed no significant differences between groups. However, the hBD-2 hydrogel group exhibited the greatest reduction in CD3-positive cells (by more than 1.15-fold) (Fig. 4F, G, I, and J). Lastly, the pattern of the pro-inflammatory IL-6 marker was also investigated, but no significant changes in expression levels were observed among the experimental groups (Fig. 4H and K). Still, the hBD-2 hydrogel group revealed a decrease by more than 1.25-fold of IL-6 expression levels compared to the other experimental groups (Fig. 4H and K).

### hBD-2 hydrogels promote proliferation and angiogenesis in skin MRSA-infected wounds of diabetic mice

**Fig. 5:**
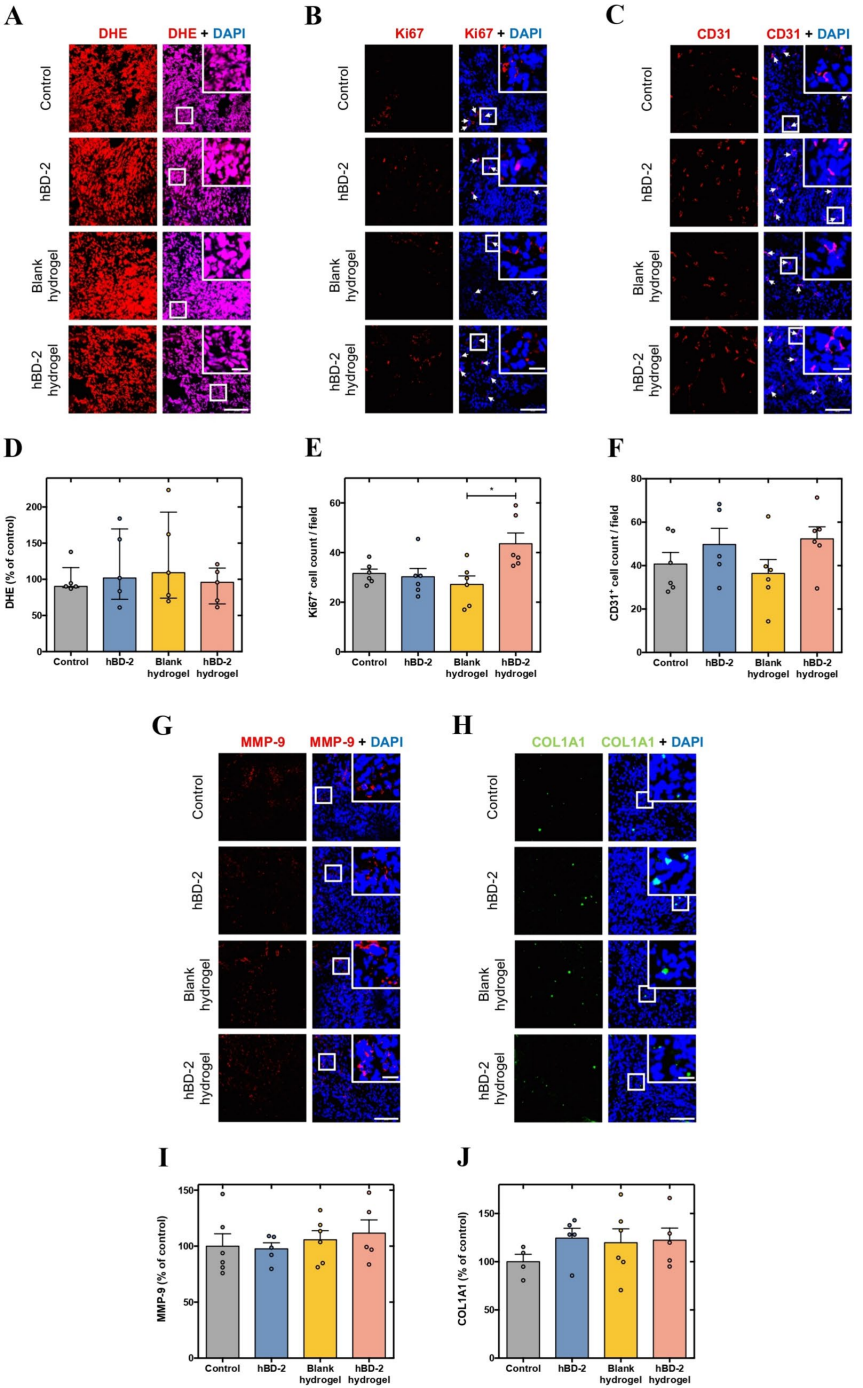
Effects of hBD-2 hydrogels on wound healing progression at day 10 post-injury/infection. Representative images of **A** dihydroethidium assay (DHE), **B** Ki67^+^ cells (indicated by white arrows), **C** CD31^+^ cells (indicated by white arrows), **G** matrix metalloproteinase-9 (MMP-9), and **H** collagen type 1 alpha 1 (COL1A1). Scale bars—20 µm (picture inlets) and 100 µm. **D** Percentage of DHE expression levels relative to the control group. **E** Number of Ki67^+^ cells per field at the wound site. **F** Number of CD31.^+^ cells per field at the wound site. **I** Percentage of MMP-9 expression levels relative to the control group. **J** Percentage of COL1A1 expression levels relative to the control group. Bars represent mean with individual measurements denoted by scatter points and the error bars indicate standard error of the mean (SEM), while non-parametric data are presented as median with interquartile range, individual measurements being denoted by scatter points. Statistical analysis was conducted using the one-way ANOVA followed by Holm–Sidak’s multiple comparisons test or the Kruskal–Wallis test followed by Dunn’s multiple comparisons test, depending on the data distribution. Asterisk specifies statistically significant differences between conditions. *p < 0.05

Additional wound healing parameters were evaluated at day 10 post-injury/infection to seek for other effects of the hBD-2 hydrogels. ROS levels were measured by DHE staining, yet no significant differences were observed among the experimental groups (Fig. 5A and D). However, hBD-2 hydrogels significantly increased the number of Ki67-positive proliferating cells compared to blank hydrogels (*p* < 0.05), and by more than 1.35-fold compared to control and free hBD-2 (Fig. 5B and E). In turn, neovascularization, determined by quantification of CD31-positive cells, was enhanced in both free hBD-2 and hBD-2 hydrogel groups by more than 1.20-fold and 1.25-fold, respectively, indicative of improved neovascularization, compared to the control and the blank hydrogel groups (Fig. 5C and F). Finally, wound remodeling and maturation were evaluated via MMP-9 and COL1A1 expression levels relative to the control group. No significant changes were observed in the content of MMP-9 between the experimental groups (Fig. 5G and I), whereas COL1A1 expression levels were yet slightly increased by more than 1.19-fold in all three free hBD-2, blank hydrogel, and hBD-2 hydrogel groups compared to the control group (Fig. 5H and J).

## Discussion

Chronic wounds, particularly those associated with diabetes mellitus, present a significant clinical challenge, due to impaired healing processes, persistent inflammation, and increased susceptibility to infections, notably by MRSA. The development of advanced therapeutic strategies that can simultaneously address microbial infection, modulate inflammation, and promote tissue regeneration is imperative. In this context, we designed alginate-based hydrogels for the sustained release of the AMP hBD-2, a peptide well-recognized for its broad-spectrum antimicrobial activity, including against the most prevalent pathogens in DFIs [6, 25]. Besides, the AMP hBD-2 also shows wound healing properties [25], thereby emphasizing the potential of this therapeutic strategy to address the multifactorial etiology of chronic DFUs [6, 11, 26]. The effects of the produced hBD-2 hydrogels on wound healing were investigated using an STZ-induced diabetic mouse model with MRSA-infected wounds.

This model was developed based on previous studies from our laboratory, where fundamental similarities were observed between the wounds from the STZ-induced diabetic mouse model and those from patients with diabetes [27]. Along with the above, the inoculation with 10^8^ CFUs/wound of MRSA for 24 h to establish the wound infection allowed a better mimic of the complexity of chronic DFUs. Indeed, more than half of DFUs may become infected, further exacerbating their harmful effects and socioeconomic burden [1]. Similarly, other studies developed diabetic rodent models of infected wound healing, yet without long-term diabetes or using lower microbial loads [18–21], underlining our animal model as a reliable option for studying complex wounds in conditions of both diabetes and infection [22]. Besides, although the establishment of infection in our model involved only a single strain, it employed a clinically relevant MRSA strain isolated from a deep lower leg wound. Furthermore, it has been reported that *Staphylococcus* is the most abundant genus detected in DFU samples, with *S. aureus* being the predominant species [1]. Moreover, *Staphylococcus* is recognized as the most frequent cause of biofilm-associated infections, and *S. aureus* is considered the most persistent species due to its virulence profile [28]. Taken together, these factors support the suitability of our model for evaluating the therapeutic potential of novel biomaterial-based approaches in the treatment of complex, infected diabetic wounds.

After topical application in the established mouse model, hBD-2 hydrogels significantly accelerated wound closure at day 7 compared to control and at day 10 compared to blank hydrogels. The hBD-2 hydrogels also induced an overall faster wound closure by more than 1.16-fold from day 3 to 7 compared to all the other experimental groups. These findings align with previous reports on β-defensins stimulating wound healing, due to their antimicrobial effects on pathogens and their stimulating effects on wound repair and immune cells [29–31], consequently enhancing the features of the hydrogels. Moreover, these results align with previous studies, indicating that AMPs like hBD-2 can promote keratinocyte proliferation and migration, essential processes in wound reepithelialization [32]. Notably, free hBD-2 showed similar results on wound closure, yet to a slighter extent, supporting a synergistic effect between the AMP hBD-2 and the hydrogel, as well as a protective effect of the alginate-based hydrogel against protease degradation and serum inactivation encountered in the host diabetic wound environment.

Moreover, our findings demonstrated that the hBD-2 hydrogels exhibited enhanced reepithelialization and early tissue remodeling, though with no significant improvement in the overall histology score. Comparable outcomes were observed with the blank hydrogels, yet to a slighter extent, suggesting that the hydrogel may act as a scaffold providing structural support for cell migration, aside from the synergistic effect between the AMP hBD-2 and the alginate-based hydrogel. Similar results were described by Winter, who outlined the use of alginate-based dressings as a scaffold for cell migration and proliferation, thereby promoting wound healing [33]. Shi et al. also demonstrated the potential of an alginate hydrogel loaded with 200 µg/mL of the AMP Chol-37(F34-R) on wound histology amelioration using a mouse model of *P. aeruginosa*-infected wounds [34]. In detail, their loaded hydrogel, yet with a considerably higher concentration of AMP than the one used in our study (1 µg/mL), exhibited better hierarchical structure between the epidermis and dermis, as well as more hair growth, compared to control, free Chol-37(F34-R) and blank hydrogel groups, thus improving wound appearance [34]. Nonetheless, the mouse model of infected wounds used by others do not consider the condition of diabetes itself, consequently not mimicking the complex microenvironment of DFUs.

Besides, the results expose that both free hBD-2 and hBD-2 hydrogels exhibited a substantial reduction in MRSA load within the wound bed compared to blank hydrogels, particularly evident by day 10 post-injury/infection. However, no significant changes on the MRSA load were observed compared to the control. These results support the antimicrobial properties of hBD-2, while also suggesting that the blank hydrogels, i.e., lacking any antimicrobial, are providing a moist environment that can be favorable for microbial growth. Although a reduction in MRSA load was also observed in the control group, this may be explained by the premature removal of Tegaderm in some animals, which likely exposed the microorganisms to air and subsequent drying, thereby preventing their growth [35]. Overall, these outcomes are potentially contributing to the resolution of the inflammatory phase, therefore fostering a better healing progression. As described above, these findings are in line with other authors that reported the beneficial impact of β-defensins on wound healing owing to their well-known antimicrobial effects on pathogens [29–31]. Moreover, Shi et al. further demonstrated that Chol-37(F34-R)-loaded alginate hydrogels inhibited the in vitro growth of *P. aeruginosa*, yet the microbial load was not assessed in the mouse model of infected wounds [34]. Despite the bacteria used in the study was isolated from a clinical case, no mention is given about the clinical condition of the patient. Thereby, this strain may not be representative of the pathogens present in the human chronic wound condition, as the levels of strains and species widely vary in the microbiome of diabetic wounds and are associated with different clinical outcomes [1]. Furthermore, the bacterial inhibition was evaluated after only 2 h of infection, which may not reflect the complex and persistent microbial burden of DFUs that largely affects healing progression. In contrast, our model allowed for pathogen establishment and interaction with the chronic diabetic milieu, offering more rigorous testing conditions. Other studies have shown that curcumin-loaded chitosan-based hydrogels accelerate healing of *S. aureus*-infected wounds in a rat model and inhibited the corresponding growth of *S. aureus*, by regulating inflammation, scavenging ROS, promoting angiogenesis, and stimulating collagen synthesis in the wound site [36]. However, similar to many such studies, the rat model with infected wounds used by others does not account for the diabetic condition nor do they use a pathogenic strain representative of chronic human wounds, therefore not mimicking the complex, chronic diabetic microenvironment.

Numerous pro-inflammatory cytokines act as key players in the recruitment of immune cells to the wound site either for clearance of cellular debris from injured skin cells or for infection control [37]. Under diabetic conditions, a persistent state of low-grade inflammation subsists with an unbalanced accumulation of immune cells and pro-inflammatory cytokines, being responsible for chronic non-healing wounds [38, 39]. Therefore, the switch from a pro- to an anti-inflammatory state is crucial for the wound healing progression to the subsequent phases [37, 40]. A critical aspect of chronic wound pathology in diabetes is the dysregulated inflammatory response, characterized by prolonged presence of pro-inflammatory M1-like macrophages and insufficient transition to the anti-inflammatory M2-like phenotype. Our results indicated that hBD-2 hydrogel treatment led to a decrease in M1-like macrophages and an increase in M2-like macrophages, resulting in a significantly lower M1/M2 ratio compared to the other experimental groups. This shift toward a pro-regenerative immune profile is crucial for resolving inflammation and facilitating tissue repair. The free hBD-2 group also showed a tendency toward an increase of anti-inflammatory M2-like macrophages, suggesting that the hydrogel protects the AMP hBD-2, conferring it a better effect. Besides, the hBD-2 hydrogels revealed a tendency toward a decrease of lymphocytes and IL-6 expression levels, but not of neutrophils, compared to the other experimental groups. Altogether, these findings corroborate that the hBD-2 hydrogel treatment stimulates a transition toward a less inflammatory but more restorative phase, triggering wound healing progression.

Elevated accumulation of ROS in the wound bed, which is characteristic in diabetic conditions, also contributes to poor healing, eliciting cell damage, compromised angiogenesis, and altered extracellular matrix remodeling [41, 42]. In our study, ROS levels remained unchanged among the experimental groups. This may be explained by the delay of the healing process owing to the presence of MRSA infection on top of the chronic diabetes condition, suggesting that healing was still in an early regenerative phase after the 10 days of the experimental period. Indeed, this aligns with other reports showing that bacterial infections in murine wounds, particularly inoculated with *P. aeruginosa* biofilms, delay wound closure and prolong inflammation, decreasing reepithelialization and collagen deposition, compared to non-infected wounds [43]. Subsequently, we looked forward for additional signs of a more reparative state. Despite unchanged ROS levels, hBD-2 hydrogels increased the number of proliferative Ki67-positive cells and both free hBD-2 and hBD-2 hydrogels showed a tendency toward an increase of CD31-positive cells, which indicate a shift toward tissue repair and neovascularization. These outcomes are of high importance for adequate reepithelialization and sufficient provision of nutrients, oxygen, and immune cells to the healing tissue [37, 38]. Similarly, other studies have reported the relevance of oxygen support in promoting tissue repair, demonstrating that self-healing hydrogels capable of delivering a continuous oxygen supply over a 10-day experimental period decreased inflammation and enhanced angiogenesis and collagen deposition in a hypoxic mouse burn model [44]. Lastly, the hBD-2 hydrogels revealed a slight tendency for an increase of COL1A1 expression levels compared to the other experimental groups, suggestive of initial remodeling. Overall, although some results did not reach statistical significance, treatment with hBD-2 hydrogels demonstrated potential biological significance. This means that the observed differences seem to have meaningful impact in wound healing progression and injury recovery. Indeed, we can observe from the wound photographs that hBD-2 hydrogels enhanced wound closure, as well as reduced inflammation in the wound bed, led to more mature histological features and attenuated MRSA infection, thereby supporting their therapeutic potential. Taken together, these findings support the evidence of an early progression toward the final phases of wound healing upon treatment with hBD-2 hydrogels.

In summary, this study demonstrates that hBD-2-loaded alginate-based hydrogels offer a multifunctional approach for treating infected diabetic wounds by combining antimicrobial action with immune modulation and regenerative support. The hydrogel matrix not only stabilizes hBD-2, but also provides a conducive microenvironment for healing. Together, these results validate the potential of AMP-loaded biomaterials in addressing the complex pathophysiology of DFUs and support further translational development of hBD-2 hydrogels as an innovative therapeutic strategy for chronic wound care.

## Conclusions

This study provides a proof-of-concept for the use of hBD-2 hydrogels for promoting both infection control and wound healing in a diabetic mouse model with MRSA-infected wounds. Although wounds remained in early stages of regeneration by 10 days, likely due to the combined burden of diabetes and infection, the findings demonstrate a significant shift toward a less inflammatory but more restorative phase upon treatment with hBD-2 hydrogels. Besides, though involving a relevant clinical strain of MRSA, the establishment of wound infection was made with a single strain, which does not fully represent the polymicrobial nature of chronic DFUs, thus denoting a limitation of this study. Correspondingly, further studies should comprise larger sample sizes and include polymicrobial biofilms to more accurately mimic the species interactions that are present in the human chronic wound condition. Nonetheless, our findings support the therapeutic potential of our newly designed hydrogel for treating chronic wound infections such as DFIs and the possible future translation into the clinic.

## Data Availability

No datasets were generated or analysed during the current study.
